# RNA-Seq-Based WGCNA and Association Analysis Reveal the Key Regulatory Module and Genes Responding to Salt Stress in Wheat Roots

**DOI:** 10.3390/plants13020274

**Published:** 2024-01-17

**Authors:** Jiating Chen, Lei Zhang, Yingxi Liu, Xinyao Shen, Yujing Guo, Xiaofei Ma, Xiaojun Zhang, Xin Li, Tianling Cheng, Huiqin Wen, Linyi Qiao, Zhijian Chang

**Affiliations:** 1 College of Agronomy, Shanxi Key Laboratory of Crop Genetics and Molecular Improvement, Shanxi Agricultural University, Taiyuan 030031, China; chenjiating111@126.com (J.C.); zxjemail@163.com (X.Z.); lixin@sxau.edu.cn (X.L.); abc7120890@sxau.edu.cn (T.C.); w7580608@sxau.edu.cn (H.W.); 2 Department of Biology, Taiyuan Normal University, Taiyuan 030031, China; lei.zhang@tynu.edu.cn; 3 Institute of Wheat Research, Shanxi Agricultural University, Linfen 041000, China

**Keywords:** wheat, salt tolerance, root, RNA-Seq, WGCNA, association analysis

## Abstract

Soil salinization is the main abiotic stressor faced by crops. An improved understanding of the transcriptional response to salt stress in roots, the organ directly exposed to a high salinity environment, can inform breeding strategies to enhance tolerance and increase crop yield. Here, RNA-sequencing was performed on the roots of salt-tolerant wheat breeding line CH7034 at 0, 1, 6, 24, and 48 h after NaCl treatment. Based on transcriptome data, a weighted gene co-expression network analysis (WGCNA) was constructed, and five gene co-expression modules were obtained, of which the blue module was correlated with the time course of salt stress at 1 and 48 h. Two GO terms containing 249 differentially expressed genes (DEGs) related to osmotic stress response and salt-stress response were enriched in the blue module. These DEGs were subsequently used for association analysis with a set of wheat germplasm resources, and the results showed that four genes, namely a Walls Are Thin 1-related gene (*TaWAT*), an aquaporin gene (*TaAQP*), a glutathione S-transfer gene (*TaGST*), and a zinc finger gene (*TaZFP*), were associated with the root salt-tolerance phenotype. Using the four candidate genes as hub genes, a co-expression network was constructed with another 20 DEGs with edge weights greater than 0.6. The network showed that *TaWAT* and *TaAQP* were mainly co-expressed with fifteen interacting DEGs 1 h after salt treatment, while *TaGST* and *TaZFP* were mainly co-expressed with five interacting DEGs 48 h after salt treatment. This study provides key modules and candidate genes for understanding the salt-stress response mechanism in wheat roots.

## 1. Introduction

Soil salinization is a universal environmental issue that results in detrimental abiotic stress in agriculture, which inhibits crop growth and reduces grain yield [1]. Approximately 1 billion hectares of land worldwide are currently salinized, representing about 7% of the Earth’s land surface and 33% of irrigated lands [2]. With the continuous growth of the global population, it is necessary to fully utilize salinized land to increase crop yields and meet the growing demand for food. As the most widely planted crop in the world, common wheat (*Triticum aestivum* L., 2n = 6x = 42; AABBDD) contributes about one-fifth of the total calories and proteins of daily dietary intake [3]. To ensure food security, it is crucial to explore salt-tolerance genes from germplasm and to utilize them to enhance the adaptability of wheat varieties in saline soil.

For common wheat, which has an enormous and complex hexaploid genome, transcriptome sequencing is a rapid and effective method for mining candidate genes [4,5,6], especially in studies involving a stress response. Based on RNA-sequencing (RNA-Seq) data, many important biological or abiotic stress response genes have been further identified, such as the leaf rust-resistance gene *Lr47* [7] and the cold tolerance gene *TaSnRK1α* [8]. RNA-Seq has also been applied in salt tolerance research. In Kharchia Local, a well-known salt-tolerant wheat germplasm resource in India, 2495 differentially expressed genes (DEGs) in the roots [9] and 3197 DEGs in the flag leaves [10] were identified by comparing the transcriptomes of seedlings in the NaCl-treated group and the control group, and 22 salt-induced unigenes were selected and validated through qRT-PCR. Similar analysis on Saudi salt-tolerant wheat germplasm resource No. 193 showed that 5829 genes were differentially expressed in the roots, whereas 3495 genes were identified in the shoots, of which eight DEGs were validated [11]. Moreover, RNA-Seq data from other salt-tolerant germplasm, such as the Chinese cultivars Qingmai 6 [12], Xiaoyan 60 [13], Jimai 22 [14], and Cangmai 6005 [15], as well as Iranian cultivar Arg [16], also revealed many DEGs responding to salt stress, helping to further identify salt-tolerance genes in wheat. However, most of the previously reported RNA-Seq analyses have been based solely on DEGs and use simple qRT-PCR to validate candidate genes, which cannot uncover more useful information. The salt tolerance of wheat is a complex quantitative trait controlled by multiple genes, making it more suitable to use network analysis methods to identify important salt-tolerance response pathways and strongly related genes.

In model plants, such as *Arabidopsis*, seedlings respond strongly at the molecular level in the early stage of salt stress. As the first organ to be exposed to stress, the roots directly face osmotic stress due to high salt concentrations in the soil [17] and suffer from salt stress, as well as the entrance of Na^+^ through a nonselective cation channel or other means [18]. Subsequently, pathways such as signal transduction and transcriptional regulation respond, initiating the plant’s salt-tolerance mechanism [19,20]. With the development of high-throughput sequencing technology, weighted gene co-expression network analysis (WGCNA) is becoming increasingly mature [21]. This method can obtain a series of biologically significant co-expression modules, and has been successfully used to analyze the transcriptome data of multiple samples in rice [22] and maize [23], identifying key regulatory pathways and genes responsive to salt stress. WGCNA was also adopted in a recent study on wheat that reported the expression patterns and the time course of root tissue from salt-tolerant wheat line ST9644 under NaCl stress [24]. Here, we performed RNA-Seq on the roots of salt-tolerant wheat breeding line CH7034 [25] after 0, 1, 6, 24, and 48 h of NaCl treatment, and performed WGCNA to screen for important gene co-expression modules related to salt stress. Then, we used salt-tolerant phenotype data from a set of wheat germplasm to perform association analyses on the genes in the selected co-expression modules to identify key salt-tolerance genes.

## 2. Results

### 2.1. RNA-Seq Data Evaluation

The transcriptome sequencing of 15 root samples of the salt-tolerant breeding line CH7034 resulted in 166.70 Gb clean reads. Each sample obtained clean data above 9.54 Gb with Q30-based percentages greater than 93.60% and GC percentages ranging from 52.06 to 54.05% (Table S1), indicating that the RNA-Seq data were of high quality for further analysis. Subsequently, 452,237,372 clean reads were mapped on the Chinese Spring reference genome RefSeq v1.0, and the uni-transcripts were annotated as known genes. A total of 56,420 genes with an average fragments per kilobase of exon model per million mapped fragments (FPKM) of >1 in at least one treatment (1, 6, 24, and 48 h) were considered expressed genes.

**Figure 1:**
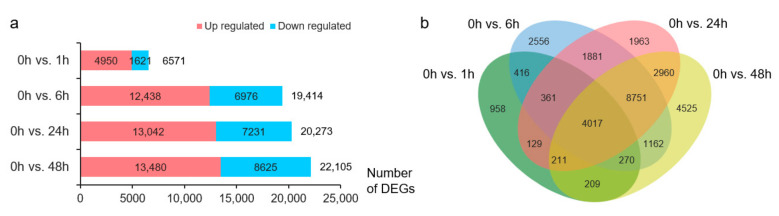
Overview of differentially expressed genes (DEGs). (**a**) Number of DEGs in each pair. Red, upregulated DEGs; blue, downregulated DEGs. (**b**) Venn diagram of the DEGs before and after NaCl treatment. Green, 0 h vs. 1 h; blue, 0 h vs. 6 h; red, 0 h vs. 24 h; yellow, 0 h vs. 48 h.

Differential expression analysis identified 6571, 19,414, 20,273, and 22,105 DEGs at 1, 6, 24, and 48 h of NaCl treatment, respectively, compared with the transcriptome data at 0 h (Figure 1a). The number of DEGs upregulated by salt stress was greater than that of downregulated DEGs (Figure 1a). Overall, 30,369 DEGs were upregulated or downregulated in at least one treatment, and 4017 DEGs appeared at each stress time point (Figure 1b).

### 2.2. Gene Co-Expression Modules Responding to Salt Stress in Roots

**Figure 2:**
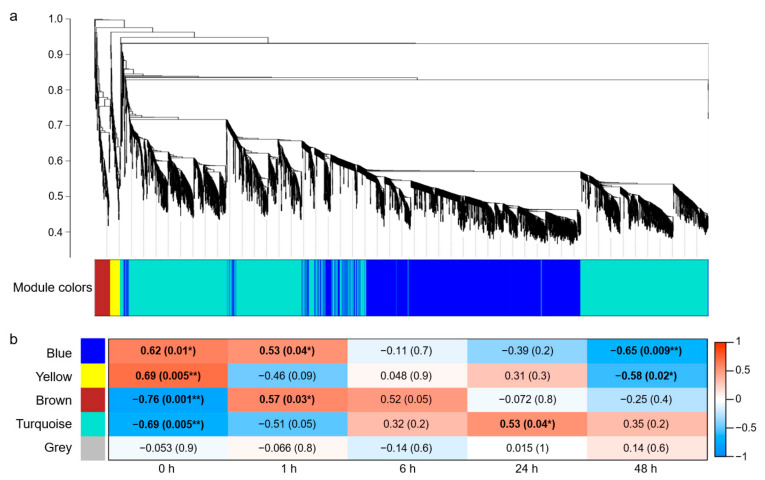
Weighted gene co-expression network analysis (WGCNA) in salt-stress response of wheat roots. (**a**) Cluster dendrogram and module colors of 4202 DEGs. (**b**) Heatmap for the relationships of modules and time courses. Each cell contains the corresponding correlation and *p*-value in parentheses. * indicates *p* < 0.05, and ** indicates *p* < 0.01.

WGCNA was performed on the 30,369 DEGs screened above, and 4202 genes were clustered and divided into five modules with different colors based on their expression patterns (Figure 2a). Among them, the gray module contained two genes that could not be assigned to any module, and the remaining four modules, namely turquoise, blue, brown, and yellow, contained 2305, 1706, 118, and 71 genes, respectively (Table S2). 

The Pearson correlation coefficient (*r* > 0.5) and the significance of the *p* value (*p* < 0.05) were used as the criteria to measure the relationship between module and time course of salt stress. The results revealed a significant correlation between the blue module and time points 0, 1, and 48 h, with “blue module—1 h” showing a positive correlation (*r* = 0.53; *p* = 0.04) and “blue module—48 h” showing a negative correlation (*r* = −0.65; *p* = 0.009). Moreover, a positive and negative correlation was shown between the yellow module and time points 0 h (*r* = 0.69; *p* = 0.005) and 48 h (*r* = −0.58; *p* = 0.02), respectively. The brown module was negatively correlated with 0 h (*r* = −0.76; *p* = 0.001) and positively correlated with 1 h (*r* = 0.57; *p* = 0.03). The turquoise module was negatively correlated with 0 h (*r* = −0.69; *p* = 0.005) and positively correlated with 24 h (*r* = 0.53; *p* = 0.04), and the gray module was not related to any time point (Figure 2b).

Compared with the other modules, the blue module was associated with more salt-treatment time points (0, 1, 48 h) and showed a strong correlation at 48 h. Therefore, all genes in the blue module were used for subsequent Gene ontology (GO) enrichment analysis.

### 2.3. The GO Enrichment of the Blue Module

**Figure 3:**
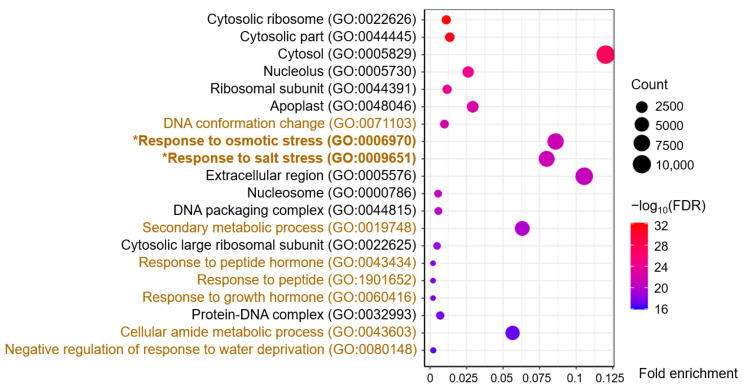
Gene ontology (GO) enrichment analysis of DEGs in the blue-module. The brown and black GO terms belong to the categories of biological process and cellular components, respectively.

A total of 455 terms were enriched from the blue module, of which 302 involved biological processes, 86 involved molecular functions, and 67 involved cellular components (Table S3). The 20 terms with the highest −log_10_ (FDR, false discovery rate) values, including 11 cellular component terms and nine biological process terms, are shown in Figure 3. The cellular component terms contained Cytosolic ribosome, Nucleolus, and Ribosomal subunit, among others, indicating a close correlation with gene transcription and protein synthesis. Furthermore, the biological process terms included Response to osmotic stress (GO:0006970) and Response to salt stress (GO:0009651), the two key GO terms related to the salt-stress response (Figure 3). Interestingly, all 236 DEGs of the GO:0009651 term were included in the 249 DEGs of the GO:0006970 term; hence, we focused on these 249 DEGs, which could be related to osmotic/salt stress (Table S4).

### 2.4. Association Analysis of the DEGs from the Key Terms

**Figure 4:**
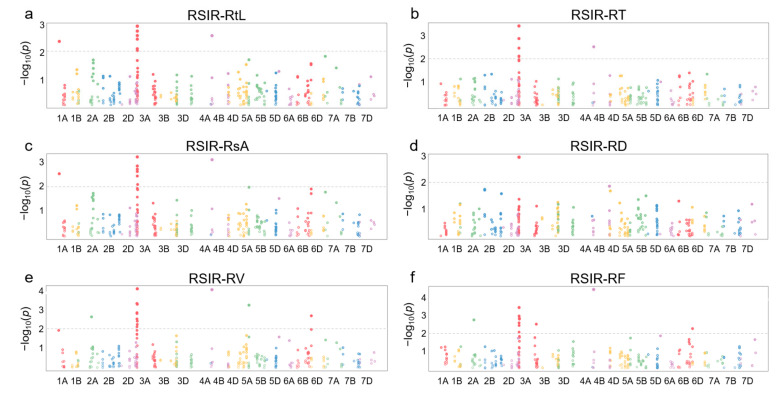
Manhattan plots for association analysis of DEGs and root salt-tolerant phenotypes in 114 wheat accessions. (**a**) Relative salt-injury rate (RSIR) for total root length (RtL). (**b**) RSIR for root tip number (RT). (**c**) RSIR for total surface area (RsA). (**d**) RSIR for average diameter (RD). (**e**) RSIR for total volume (RV). (**f**) RSIR for root branching number (RF).

Information on 368 SNPs from 120 of 249 DEGs in 114 wheat germplasm resources was obtained from the WheatUnion database (Table S5). For the involved wheat germplasm, six salt-tolerant phenotypes associated with roots, including total root length (RtL), root tip number (RT), total surface area (RsA), average diameter (RD), total volume (RV), and root branching number (RF), were identified, and the relative salt-injury rates (RSIR) were calculated based on data from the control group and NaCl treatment group. Association analysis results showed that 47 SNPs distributed on chromosomes 1A, 2A, 3A, 4B, 5B, and 6B were correlated with salt-tolerance phenotypes in roots (*p* < 0.01) (Figure 4).

After further screening, four high-confidence SNPs were selected, including 1A337654393, 3A7437277, 5B91533091, and 6B708989259, with a minimum allele frequency > 10% and a maximum missing rate of <5% (Table S6). Among them, 1A337654393 from *TraesCS1A02G186300* was associated with RSIR-RtL (*p* = 0.0043) and RSIR-RsA (*p* = 0.003), and 3A7437277 from *TraesCS3A02G007100* was associated with RSIR-RtL (*p* = 0.0018), RSIR-RsA (*p* = 0.0024), RSIR-RT (*p* = 0.0076), and RSIR-RV (*p* = 0.0076). SNP 5B91533091 from *TraesCS5B02G076400* and 6B708989259 from *TraesCS6B02G450000* were both associated with RSIR-RV (*p* = 0.0006 and *p* = 0.002, respectively) (Table S6).

### 2.5. Candidate Genes Responding to Salt Stress in Roots

**Figure 5:**
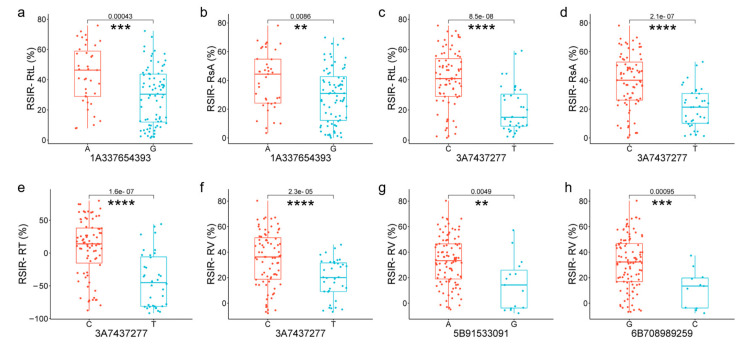
Significant differences analysis in genotypes of high-confidence SNPs from four candidate genes. (**a**,**b**) Differences in RSIR-RtL and -RsA corresponding to genotypes of 1A337654393(A/G) of *TaGST*. (**c**–**f**) Differences in RSIR-RtL, -RsA, -RT, and -RV corresponding to genotypes of 3A7437277(C/T) of *TaWAT*. (**g**) Differences in RSIR-RV corresponding to genotypes of 5B91533091(A/G) of *TaZFP*. (**h**) Differences in RSIR-RV corresponding to genotypes of 6B708989259(G/C) of *TaAQP*. Red and blue represent base allele variations of SNP. ** indicates *p* < 0.01, *** indicates *p* < 0.001, and **** indicates *p* < 0.0001.

Among the four candidate genes, *TraesCS1A02G186300* encoded glutathione S-transferase (*TaGST*). SNP 1A337654393, located in exon 2 of *TaGST,* changed A to G, causing the amino acid Leucine (L) to change to Proline (P), decreasing RSIR-RtL (*p* = 0.00043, Figure 5a) and RSIR-RsA (*p* = 0.0086, Figure 5b). *TraesCS3A02G007100* encoded the Walls Are Thin 1 (WAT1)-related protein (*TaWAT*). SNP 3A7437277 (C/T), located in exon 2 of *TaWAT,* caused amino acids to change from Glycine (G) to Serine (S), decreasing RSIR-RtL (*p* = 8.5 × 10^−8^, Figure 5c), RSIR-RsA (*p* = 2.1 × 10^−7^, Figure 5d), RSIR-RT (*p* = 1.6 × 10^−7^, Figure 5e), and RSIR-RV (*p* = 2.3 × 10^−5^, Figure 5f). *TraesCS5B02G076400* with no intron encoded a putative zinc finger protein (*TaZFP*). SNP 5B91533091(A/G) in *TaZFP* caused amino acid S to change to G, decreasing RSIR-RV (*p* = 0.0049, Figure 5g), and *TraesCS6B02G450000* encoded Aquaporin (*TaAQP*). SNP 6B708989259(G/C), located in exon 3 of *TaAQP*, changed the amino acid Alanine (A) to *p*, which was associated with the decrease in RSIR-RV (*p* = 0.00095, Figure 5h).

### 2.6. Gene Co-Expression Networks Responding to Short-Term Salt Stress in Roots

**Figure 6:**
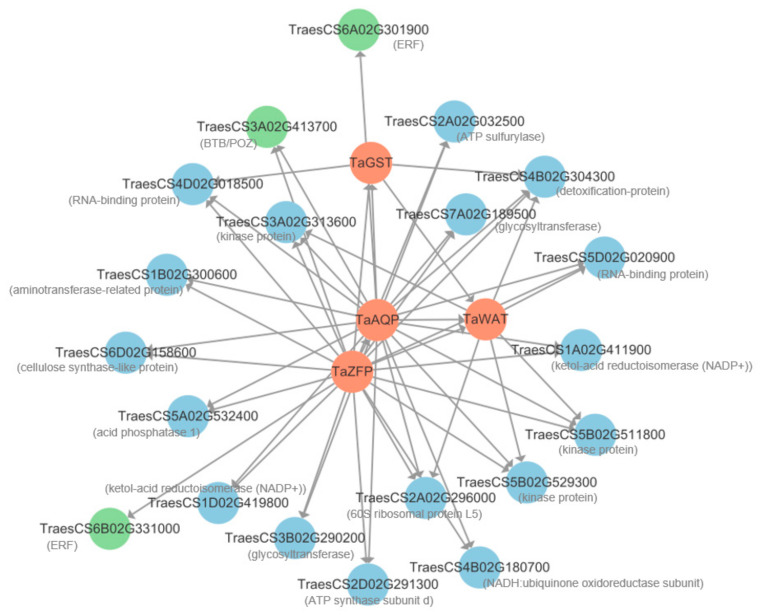
Co-expression network for four hub genes and 20 DEGs with the highest edge weight values from selected terms in the blue module. The red, green, and blue circles represent hub-genes, regulatory genes, and structural genes, respectively. Arrows indicate the direction of interaction.

Twenty genes with the highest edge weight co-expressed with the four candidate genes were selected from the 249 DEGs to construct an interaction network. The results showed that 3, 6, 21, and 22 genes interacted with *TaGST*, *TaWAT*, *TaAQP*, and *TaZFP*, respectively (Figure 6, Table S7). These interacting genes were divided into two categories. One category contained regulatory genes, including *TraesCS3A02G413700* encoding Broad Complex, Tramtrack, and Bric-A-Brac (BTB)/POX Virus and Zinc Finger (POZ) protein, and *TraesCS6A02G301900* and *TraesCS6B02G331000*, encoding ethylene-responsive factor (ERF). The other category contained the following structural genes: *TraesCS3A02G313600*, *TraesCS5B02G511800*, and *TraesCS5B02G529300*, encoding kinase; *TraesCS3B02G290200* and *TraesCS7A02G189500*, encoding glycosyltransferase; and *TraesCS4B02G304300*, encoding a detoxification protein (Table S7).

**Figure 7:**
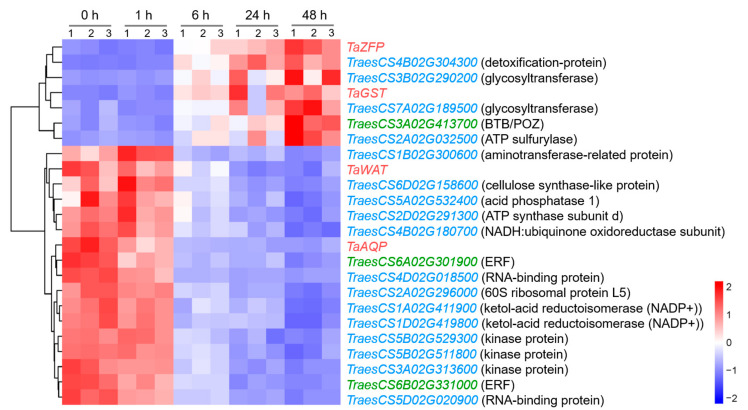
Heatmap of genes in the co-expression network at 0, 1, 6, 24, and 48 h after salt stress in wheat root. The red, green, and blue genes represent hub-genes, regulatory genes, and structural genes, respectively, and the gene annotations based on Chinese Spring RefSeq v1.1 are listed in parentheses.

Among the genes in this network, 17 genes, including *TaWAT* and *TaAQP*, were upregulated in the early stage of NaCl treatment, while the remaining seven genes, including *TaZFP* and *TaGST*, were upregulated in the later stages of stress, mainly at 48 h (Figure 7).

## 3. Discussion

### 3.1. The Blue Module Is Related to the Time Course of Salt-Stress Response in Wheat Roots

The roots are the first organ exposed to high salt stress. Exploring the key modules and genes in roots is crucial not only for a deeper understanding of salt-tolerance mechanisms but also for salt-tolerance molecular breeding in wheat. Research in model plants has shown that cellular responses can be placed in different phases after salt treatment [26]. Early signaling represents changes observed within 5 min of salt stress application in the roots. The transcriptional response from the nucleus is activated, and the growth rate decreases during the stop phase and the quiescent phase within 5 min to 5 h and 5–9 h of salt application, respectively. Later, plants partially recover their growth rate during the growth recovery phase after 9 h of salt application [26].

In this study, we set four time points for the NaCl treatment of roots: 1 h during the stop phase, 6 h during the quiescent phase, and 24 and 48 h during the growth recovery phase. The RNA-Seq results showed that, compared with the untreated control, the number of DEGs increased with the prolongation of treatment time, and there were more upregulated genes than downregulated genes. These DEGs were divided into five modules through WGCNA, of which the blue module was significantly positively correlated with 0 and 1 h and significantly negatively correlated with 48 h, indicating that this module was related to the time course and primarily functioned during the stop and growth recovery phases. Transcriptional changes occurring in the stop phase promote the synthesis of functional proteins, such as biochemical enzymes and ion transporters [27], and activate stress-related ABA or ethylene signaling pathways [19,28]. In the later growth recovery phase, genes involved in growth repair begin to be regulated [29].

### 3.2. Four Novel Candidate Genes Responding to Salt Stress Discovered from the Blue Module

Two biological process terms, Response to osmotic stress (GO:0006970) and Response to salt stress (GO:0009651), were enriched in the blue module. Of 249 DEGs, 4, namely *TaWAT*, *TaAQP*, *TaZFP*, and *TaGST*, from the two GO terms were significantly correlated with salt-tolerance phenotypes in the roots using a set of wheat germplasm.

*TaWAT* and *TaAQP* were upregulated in the roots of CH7034 at 1 h of salt treatment. The *WAT1* gene encoding the transmembrane protein was involved in auxin transport and homeostasis [30], and was significantly upregulated by salinity stress [31,32]. In this study, *TaWAT* was associated with RSIR-RtL, -RsA, -RT, and -RV, indicating that this gene may ultimately impact the ability of roots to transport auxin and alter growth under salt application, as predicted in another study [33]. Aquaporins located on the cell plasma membrane mediate the regulation of root hydraulic conductivity in response to osmotic stress and salt stress [17,34]. The overexpression of two wheat *AQP* genes, *TaNIP* (GenBank No. DQ530420) [35] and *TaAQP8* (GenBank No. HQ650110) [36], significantly enhanced salt tolerance in the transgenic plants of *Arabidopsis* and tobacco, respectively. The upregulation of *TaAQP8* under salt stress involved an ethylene signaling pathway, causing a positive effect and then increased root elongation [36]. Moreover, a recent study reported that the AQP activity of wheat varied with the diurnal cycle, which periodically affected the plant’s tolerance to salt stress [37]. In our case, a novel *TaAQP* gene was associated with RSIR-RV, and exhibited similar expression patterns 24 and 48 h after salt treatment (Figure 7).

*TaZFP* and *TaGST* were mainly upregulated in CH7034 roots 48 h after salt treatment during the growth recovery phase. ZFP, located in the nucleus, is a type of well-studied transcription factor related to salt stress responsiveness [38,39]. *ZFP* overexpression significantly increased the levels of ascorbic acid (AsA) in *Arabidopsis* and tomato, which strengthened the AsA-mediated reactive oxygen species (ROS)-scavenging capacity and enhanced the salt tolerance of the plants [40]. In salt-tolerant wheat cultivar Shanrong No. 3, the *ZFP* gene *TaCHP* (GenBank No. GQ379226) was expressed in the roots of seedlings at the three-leaf stage, and its transcript was localized in the cells of the root tip cortex and meristem [41]. The *TaZFP* reported in this study is a novel gene associated with RSIR-RV, and the other gene, *TaGST*, is related to RSIR-RtL and RSIR-RsA. Overexpressing *GST* in cotton or soybean improves their salt tolerance [42,43,44]. In addition, GST is involved in scavenging ROS induced by salt stress in cells [45]. Therefore, *TaGST* and *TaZFP* may recover cell growth by alleviating oxidative stress, thereby enhancing the roots’ tolerance to salt stress.

### 3.3. A Predicted Gene Co-Expression Network for Salt Tolerance

Based on edge weight values, an interaction network was predicted, setting the four candidate genes as hub genes. After salt stress application to roots, *TaWAT* and *TaAQP* were upregulated and promoted the expression of multiple genes involving biochemical reactions. Some of these genes were coding kinase proteins. In the salt-stress response in *Arabidopsis*, kinase proteins play a crucial role in ion transport and in signal transduction [46,47]. Some genes referred to transcription and translation, such as the 60S ribosomal protein L5 (RPL-5), ATP synthase subunit b (ATP5H), and RNA-binding protein (RBP), which are related to salt tolerance in rice or cotton [42,43,44], and some genes referred to the metabolic processes in response to salt stress [48,49], such as the aminotransferase-related protein gene and ketol-acid reductoisomerase NADP(+) (KARI). In addition, two *ERF* genes, *TraesCS6A02G301900* and *TraesCS6B02G331000*, were also induced. Currently, three salt stress-response *ERF* genes have been identified in wheat, including two salt-tolerant genes, *TaERF1* [50] and *TaERF3* [51], as well as one salt-sensitive gene, *TaERF4* [52]. The two *ERF*s identified in this study are novel genes related to salt-stress responsiveness.

In the 6 to 48 h after salt stress, *TaZFP* and *TaGST* gene activities gradually increased, and multiple genes were induced. *TraesCS4B02G304300* encodes a detoxification protein that may alleviate cation toxicity in cells [53,54], and *TraesCS2A02G032500* encodes ATP sulfurylase that can chelate SO_4_^2−^ under salt stress [55]. *TraesCS3A02G413700* encodes a BTB/POZ transcription factor, and *TraesCS3B02G290200* and *TraesCS7A02G189500* encode glycosyltransferases. Both BTB/POZ and glycosyltransferases have been reported to scavenge ROS, which are induced by salt stress [56,57,58]. Therefore, the genes that respond during this period may primarily perform growth recovery functions under salt stress.

However, the blue module was significantly negatively correlated with the 48 h after the salt-stress time course. One possible explanation is that some genes, especially the hub genes *TaZFP* and/or *TaGST*, exist as negative regulatory factors in certain salt-tolerance pathways. Similar results have been reported. Several ZFP genes, such as *MtZPT2-2* in *Medicago truncatula* [59], *AZF1* and *AZF2* in *Arabidopsis* [60], and *DST* [61] and *OsZFP185* [62] in rice, can negatively regulate plant salt tolerance by inhibiting the ABA signaling pathway or transport proteins. Moreover, the GST gene *AtGSTU17* has also been shown to play a negative role in salt stress tolerance in *Arabidopsis* [63]. Further experiments are needed to confirm this speculation.

## 4. Materials and Methods

### 4.1. Plant Materials and Salt Treatment

Wheat breeding line CH7034 with salt tolerance was used for RNA-Seq and qRT-PCR. A set of wheat germplasm containing 114 varieties (Table S8) was used for salt-tolerance phenotype identification and association analysis.

Sterilized seeds were laid on Petri dishes covered with moist filter paper for 2–3 days. The uniform germinated seeds were then selected and transferred to half-strength Hoagland’s culture solution in a growth chamber under a 22/16 °C (day/night) temperature regime and a 16/8 h (light/dark) photoperiod with 60% relative humidity. When the seedlings grew to the three-leaf stage, they were exposed to 250 mM NaCl for salt-stress treatment.

### 4.2. RNA-Seq

The root samples of the CH7034 seedlings were collected with three replications after 0, 1, 6, 24, and 48 h of NaCl stress, then frozen in liquid nitrogen, and stored at −80 °C. Total RNA was extracted by TRIzol reagent (Invitrogen, Carlsbad, CA, USA) according to the manufacturer’s instructions, and the cDNA libraries were constructed using TruSeq RNA Sample Preparation Kit v2 (Illumina, San Diego, CA, USA). RNA-Seq was performed on all libraries by Biomarker Technologies Co., Ltd. (Beijing, China) using the HiSeq 4000 platform (Illumina, San Diego, CA, USA). Clean reads were obtained by removing low-quality reads containing adapters or poly-N from the raw data and mapped to the Chinese Spring reference genome RefSeq v1.0 (http://wheat-urgi.ver-sailles.inra.fr/, accessed on 10 March 2023). The transcript level of each gene was measured with FPKM and then normalized by log2. Differential expression analysis between the control (0 h of NaCl treatment) and salt-stress groups (1, 6, 24, and 48 h of NaCl treatment) was performed using DESeq R package [64], with a threshold of |log2FoldChange| ≥ 1 and FDR ≤ 0.01. 

### 4.3. Co-Expression Network Construction

Gene co-expression networks were constructed using R (version 4.3.1) software and the WGCNA package (version 1.72) for the RNA-Seq data from the roots. The transcript levels of all of the DEGs were converted into a similarity matrix and transformed to a topological overlap matrix using a parameter β value of 12. Genes with similar expression patterns were categorized into different modules using a bottom-up algorithm with a module minimum size cutoff of 30.

The correlation between module eigengenes and the time points for salt stress was calculated using a Pearson test, and the individual modules with *p* < 0.05 were considered significantly correlated with the time course. Then, the module with the highest correlation coefficient was used for GO enrichment analysis on the BMKCloud platform (www.biocloud.net, accessed on 24 November 2023), and items with a high correlation with salt tolerance were selected. Genes with a WGCNA edge weight > 0.6 in the selected items were visualized using Cytoscape 3.7.2 software.

### 4.4. Association Analysis

RSIR-R was investigated for 114 wheat germplasm resources. Salt stress was imposed for germplasm according to the method described in Section 4.1. A Root Scanner (MicroTek, Shanghai, China) was used to scan the wheat roots after 7 days of H_2_O treatment (CK) and NaCl treatment to obtain the total root length (RtL), total surface area (RsA), total volume (RV), average diameter (RD), root tip number (RT), and root branching number (RF). The relative salt-injury rate of each root phenotype for wheat variety was calculated using the following formula: RSIR-R (%) = (*X*_CK_ − *X*_NaCl_)/*X*_CK_ × 100%.

Information on sequence variation in the gene coding region was downloaded from the WheatUnion database (http://wheat.cau.edu.cn/WheatUnion/, accessed on 24 November 2023). Using 114 wheat germplasm resources, the correlation between the root-relative salt-injury rate data and the genes screened by WGCNA, the edge weight was evaluated using association analysis in the R (version 4.3.1) software with the GAPIT package (version 3). The correlation was considered significant when −log_10_(*p*) > 2 (i.e., *p* < 0.01).

### 4.5. Statistical Analysis

The Origin software (version 3.1) was used to perform the statistical analysis by one-way analysis of variance (ANOVA), and *p* < 0.05 was considered a statistically significant difference.

## 5. Conclusions

Based on RNA-sequencing data performed for the roots of salt-tolerant wheat breeding line CH7034 at 0, 1, 6, 24, and 48 h after NaCl treatment, we conducted WGCNA and obtained five gene co-expression modules, of which the blue module was correlated with the time course of salt stress at 1 and 48 h. Two GO terms containing 249 DEGs related to osmotic stress response and salt-stress response were enriched in the blue module, and 4 DEGs, including *TaWAT*, *TaAQP*, *TaGST*, and *TaZFP*, were associated with the root salt-tolerance phenotype in a set of wheat germplasm resources. A co-expression network constructed with the four candidate genes and another 20 DEGs showed that *TaWAT* and *TaAQP* were mainly co-expressed with 15 interacting DEGs 1 h after salt treatment, while *TaGST* and *TaZFP* were mainly co-expressed with 5 interacting DEGs 48 h after salt treatment.

## Data Availability

Data are contained within the article and Supplementary Materials.

## Supplementary Materials

The following supporting information can be downloaded at: https://www.mdpi.com/article/10.3390/plants13020274/s1, Table S1: Summary of RNA-sequencing results, Table S2: The modules and 4202 clustered genes in WGCNA, Table S3: GO enrichment of the blue-module, Table S4: 249 DEGs of GO terms related osmotic and/or salt-stress response in the blue module, Table S5: Information on 368 SNPs of DEGs used for association analysis, Table S6: Four high-confidence SNPs associated with root salt-tolerant phenotypes, Table S7: Twenty genes with the highest edge weight co-expressed with the four candidate genes, Table S8: 114 wheat germplasms used in association analysis.

## References

[1] Hassani A., Azapagic A., Shokri N. (2021). Global predictions of primary soil salinization under changing climate in the 21st century. Nat. Commun..

[2] Hopmans J.W., Qureshi A.S., Kisekka I., Munns R., Grattan S.R., Rengasamy P., Ben-Gal A., Assouline S., Javaux M., Minhas P.S., Sparks D.L. (2021). Chapter One—Critical knowledge gaps and research priorities in global soil salinity. Advances in Agronomy.

[3] Shewry P.R., Hey S.J. (2015). The contribution of wheat to human diet and health. Food Energy Secur..

[4] Wang Z., Gerstein M., Snyder M. (2009). RNA-Seq: A revolutionary tool for transcriptomics. Nat. Rev. Genet..

[5] Yang G., Pan W., Cao R., Guo Q., Cheng Y., Zhao Q., Cui L., Nie X. (2022). Multi-omics reveals the key and specific miRNA-mRNA modules underlying salt tolerance in wild emmer wheat (*Triticum dicoccoides* L.). BMC Genom..

[6] Pfeifer M., Kugler K.G., Sandve S.R., Zhan B., Rudi H., Hvidsten T.R., Mayer K.F., Olsen O.A., International Wheat Genome Sequencing Consortium (2014). Genome interplay in the grain transcriptome of hexaploid bread wheat. Science.

[7] Li H., Hua L., Zhao S., Hao M., Song R., Pang S., Liu Y., Chen H., Zhang W., Shen T. (2023). Cloning of the wheat leaf rust resistance gene *Lr47* introgressed from *Aegilops speltoides*. Nat. Commun..

[8] Zhang L., Zhang N., Wang S., Tian H., Liu L., Pei D., Yu X., Zhao L., Chen F. (2023). A TaSnRK1α modulates TaPAP6L-mediated wheat cold tolerance through regulating endogenous jasmonic acid. Adv. Sci..

[9] Goyal E., Amit S.K., Singh R.S., Mahato A.K., Chand S., Kanika K. (2016). Transcriptome profiling of the salt-stress response in *Triticum aestivum* cv. Kharchia Local. Sci. Rep..

[10] Mahajan M.M., Goyal E., Singh A.K., Gaikwad K., Kanika K. (2017). Transcriptome dynamics provide insights into long-term salinity stress tolerance in *Triticum aestivum* cv. Kharchia Local. Plant Physiol. Biochem..

[11] Alyahya N., Taybi T. (2023). Comparative transcriptomic profiling reveals differentially expressed genes and important related metabolic pathways in shoots and roots of a Saudi wheat cultivar (Najran) under salinity stress. Front. Plant Sci..

[12] Zhang Y., Liu Z., Khan A.A., Lin Q., Han Y., Mu P., Liu Y., Zhang H., Li L., Meng X. (2016). Expression partitioning of homeologs and tandem duplications contribute to salt tolerance in wheat (*Triticum aestivum* L.). Sci. Rep..

[13] Luo Q., Teng W., Fang S., Li H., Li B., Chu J., Li Z., Zheng Q. (2019). Transcriptome analysis of salt-stress response in three seedling tissues of common wheat. Crop J..

[14] Dugasa M., Feng X., Wang N., Wang J., Wu F. (2021). Comparative transcriptome and tolerance mechanism analysis in the two contrasting wheat (*Triticum aestivum* L.) cultivars in response to drought and salinity stresses. Plant Growth Regul..

[15] Wang W., Cao J., Huang S., Wang Z., Wang W., Zou J., Wang F., Luo M., Zhang J. (2023). Integrated transcriptomics and metabolomics analyses provide insights into salt-stress response in germination and seedling stage of wheat (*Triticum aestivum* L.). Curr. Plant Biol..

[16] Amirbakhtiar N., Ismaili A., Ghaffari M.R., Mirdar Mansuri R., Sanjari S., Shobbar Z.S. (2021). Transcriptome analysis of bread wheat leaves in response to salt stress. PLoS ONE.

[17] Boursiac Y., Chen S., Luu D.T., Sorieul M., van den Dries N., Maurel C. (2005). Early effects of salinity on water transport in Arabidopsis roots. Molecular and cellular features of aquaporin expression. Plant Physiol..

[18] Demidchik V., Tester M. (2002). Sodium fluxes through nonselective cation channels in the plasma membrane of protoplasts from Arabidopsis roots. Plant Physiol..

[19] Duan L., Dietrich D., Ng C.H., Chan P.M., Bhalerao R., Bennett M.J., Dinneny J.R. (2013). Endodermal ABA signaling promotes lateral root quiescence during salt stress in Arabidopsis seedlings. Plant Cell..

[20] Choi W.G., Toyota M., Kim S.H., Hilleary R., Gilroy S. (2014). Salt stress-induced Ca^2+^ waves are associated with rapid, long-distance root-to-shoot signaling in plants. Proc. Natl. Acad. Sci. USA.

[21] Langfelder P., Horvath S. (2008). WGCNA: An R package for weighted correlation network analysis. BMC Bioinform..

[22] Zhu M., Xie H., Wei X., Dossa K., Yu Y., Hui S., Tang G., Zeng X., Yu Y., Hu P. (2019). WGCNA analysis of salt-responsive core transcriptome identifies novel hub genes in rice. Genes.

[23] Ma L., Zhang M., Chen J., Qing C., He S., Zou C., Yuan G., Yang C., Peng H., Pan G. (2021). GWAS and WGCNA uncover hub genes controlling salt tolerance in maize (*Zea mays* L.) seedlings. Theor. Appl. Genet..

[24] Li J., Gao X., Chen X., Fan Z., Zhang Y., Wang Z., Shi J., Wang C., Zhang H., Wang L. (2023). Comparative transcriptome responses of leaf and root tissues to salt stress in wheat strains with different salinity tolerances. Front. Genet..

[25] Qiao L., Zhang X., Li X., Yang Z., Li R., Jia J., Yan L., Chang Z. (2022). Genetic incorporation of genes for the optimal plant architecture in common wheat. Mol. Breed..

[26] Van Zelm E., Zhang Y., Testerink C. (2020). Salt tolerance mechanisms of plants. Annu. Rev. Plant Biol..

[27] Maathuis F.J. (2006). The role of monovalent cation transporters in plant responses to salinity. J. Exp. Bot..

[28] Dou L., He K., Higaki T., Wang X., Mao T. (2018). Ethylene signaling modulates cortical microtubule reassembly in response to salt Stress. Plant Physiol..

[29] Geng Y., Wu R., Wee C.W., Xie F., Wei X., Chan P.M., Tham C., Duan L., Dinneny J.R. (2013). A spatio-temporal understanding of growth regulation during the salt stress response in Arabidopsis. Plant Cell.

[30] Ranocha P., Dima O., Nagy R., Felten J., Corratgé-Faillie C., Novák O., Morreel K., Lacombe B., Martinez Y., Pfrunder S. (2013). Arabidopsis WAT1 is a vacuolar auxin transport facilitator required for auxin homoeostasis. Nat. Commun..

[31] Zhang H., Xu W., Chen H., Chen J., Liu X., Chen X., Yang S. (2021). Transcriptomic analysis of salt tolerance-associated genes and diversity analysis using indel markers in yardlong bean (*Vigna unguiculata* ssp. sesquipedialis). BMC Genom. Data.

[32] Carvalho da Silva T.L., Belo Silva V.N., Braga Í.O., Rodrigues Neto J.C., Leão A.P., Ribeiro J.A.A., Valadares L.F., Abdelnur P.V., de Sousa C.A.F., Souza M.T. (2022). Integration of metabolomics and transcriptomics data to further characterize *Gliricidia sepium* (Jacq.) Kunth under high salinity stress. Plant Genome.

[33] Irizarry I., White J.F. (2018). *Bacillus amyloliquefaciens* alters gene expression, ROS production and lignin synthesis in cotton seedling roots. J. Appl. Microbiol..

[34] Domec J.C., King J.S., Carmichael M.J., Overby A.T., Wortemann R., Smith W.K., Miao G., Noormets A., Johnson D.M. (2021). Aquaporins, and not changes in root structure, provide new insights into physiological responses to drought, flooding, and salinity. J. Exp. Bot..

[35] Gao Z., He X., Zhao B., Zhou C., Liang Y., Ge R., Shen Y., Huang Z. (2010). Overexpressing a putative aquaporin gene from wheat, *TaNIP*, enhances salt tolerance in transgenic Arabidopsis. Plant Cell Physiol..

[36] Hu W., Yuan Q., Wang Y., Cai R., Deng X., Wang J., Zhou S., Chen M., Chen L., Huang C. (2012). Overexpression of a wheat aquaporin gene, *TaAQP8*, enhances salt stress tolerance in transgenic tobacco. Plant Cell Physiol..

[37] Lu Y., Fricke W. (2023). Changes in root hydraulic conductivity in wheat (*Triticum aestivum* L.) in response to salt stress and day/night can best be explained through altered activity of aquaporins. Plant Cell Environ..

[38] Mukhopadhyay A., Vij S., Tyagi A.K. (2004). Overexpression of a zinc-finger protein gene from rice confers tolerance to cold, dehydration, and salt stress in transgenic tobacco. Proc. Natl. Acad. Sci. USA.

[39] Sakamoto H., Maruyama K., Sakuma Y., Meshi T., Iwabuchi M., Shinozaki K., Yamaguchi-Shinozaki K. (2004). Arabidopsis Cys2/His2-type zinc-finger proteins function as transcription repressors under drought, cold, and high-salinity stress conditions. Plant Physiol..

[40] Li Y., Chu Z., Luo J., Zhou Y., Cai Y., Lu Y., Xia J., Kuang H., Ye Z., Ouyang B. (2018). The C2H2 zinc-finger protein SlZF3 regulates AsA synthesis and salt tolerance by interacting with CSN5B. Plant Biotechnol. J..

[41] Li C., Lv J., Zhao X., Ai X., Zhu X., Wang M., Zhao S., Xia G. (2010). TaCHP: A wheat zinc finger protein gene down-regulated by abscisic acid and salinity stress plays a positive role in stress tolerance. Plant Physiol..

[42] Light G.G., Mahan J.R., Roxas V.P., Allen R.D. (2005). Transgenic cotton (*Gossypium hirsutum* L.) seedlings expressing a tobacco glutathione S-transferase fail to provide improved stress tolerance. Planta.

[43] Dong Y., Li C., Zhang Y., He Q., Daud M.K., Chen J., Zhu S. (2016). Glutathione S-transferase gene family in *Gossypium raimondii* and *G. arboreum*: Comparative genomic study and their expression under salt stress. Front. Plant Sci..

[44] Li X., Pang Y., Zhong Y., Cai Z., Ma Q., Wen K., Nian H. (2023). *GmGSTU23* encoding a Tau class glutathione S-transferase protein enhances the salt tolerance of soybean (*Glycine max* L.). Int. J. Mol. Sci..

[45] Le Martret B., Poage M., Shiel K., Nugent G.D., Dix P.J. (2011). Tobacco chloroplast transformants expressing genes encoding dehydroascorbate reductase, glutathione reductase, and glutathione-S-transferase, exhibit altered anti-oxidant metabolism and improved abiotic stress tolerance. Plant Biotechnol. J..

[46] Yin X., Xia Y., Xie Q., Cao Y., Wang Z., Hao G., Song J., Zhou Y., Jiang X. (2020). The protein kinase complex CBL10-CIPK8-SOS1 functions in Arabidopsis to regulate salt tolerance. J. Exp. Bot..

[47] Chen X., Ding Y., Yang Y., Song C., Wang B., Yang S., Guo Y., Gong Z. (2021). Protein kinases in plant responses to drought, salt, and cold stress. J. Integr. Plant Biol..

[48] Yang L., Han H., Liu M., Zuo Z., Zhou K., Lü J., Zhu Y., Bai Y., Wang Y. (2023). Overexpression of the Arabidopsis photorespiratory pathway gene, serine: Glyoxylate aminotransferase (*AtAGT1*), leads to salt stress tolerance in transgenic duckweed (*Lemna minor*). Plant Cell Tissue Organ Cult..

[49] Guo Y., Song Y., Zheng H., Zhang Y., Guo J., Sui N. (2018). NADP-malate dehydrogenase of sweet sorghum improves salt tolerance of *Arabidopsis thaliana*. J. Agric. Food Chem..

[50] Xu Z.S., Xia L.Q., Chen M., Cheng X.G., Zhang R.Y., Li L.C., Zhao Y.X., Lu Y., Ni Z.Y., Liu L. (2007). Isolation and molecular characterization of the *Triticum aestivum* L. ethylene-responsive factor 1 (TaERF1) that increases multiple stress tolerance. Plant Mol. Biol..

[51] Rong W., Qi L., Wang A., Ye X., Du L., Liang H., Xin Z., Zhang Z. (2014). The ERF transcription factor TaERF3 promotes tolerance to salt and drought stresses in wheat. Plant Biotechnol. J..

[52] Dong W., Ai X., Xu F., Quan T., Liu S., Xia G. (2012). Isolation and characterization of a bread wheat salinity responsive ERF transcription factor. Gene.

[53] Gaxiola R.A., Rao R., Sherman A., Grisafi P., Alper S.L., Fink G.R. (1999). The *Arabidopsis thaliana* proton transporters, AtNhx1 and Avp1, can function in cation detoxification in yeast. Proc. Natl. Acad. Sci. USA.

[54] Tsugane K., Kobayashi K., Niwa Y., Ohba Y., Wada K., Kobayashi H. (1999). A recessive Arabidopsis mutant that grows photoautotrophically under salt stress shows enhanced active oxygen detoxification. Plant Cell.

[55] Anjum N.A., Gill R., Kaushik M., Hasanuzzaman M., Pereira E., Ahmad I., Tuteja N., Gill S.S. (2015). ATP-sulfurylase, sulfur-compounds, and plant stress tolerance. Front. Plant Sci..

[56] Li P., Li Y.J., Zhang F.J., Zhang G.Z., Jiang X.Y., Yu H.M., Hou B.K. (2017). The Arabidopsis UDP-glycosyltransferases UGT79B2 and UGT79B3, contribute to cold, salt and drought stress tolerance via modulating anthocyanin accumulation. Plant J..

[57] Wan X., Peng L., Xiong J., Li X., Wang J., Li X., Yang Y. (2019). AtSIBP1, a novel BTB domain-containing protein, positively regulates salt signaling in *Arabidopsis thaliana*. Plants.

[58] Wang T., Ma Y., Huang X., Mu T., Li Y., Li X., Liu X., Hou B. (2021). Overexpression of *OsUGT3* enhances drought and salt tolerance through modulating ABA synthesis and scavenging ROS in rice. Environ. Exp. Bot..

[59] Huang R., Jiang S., Dai M., Shi H., Zhu H., Guo Z. (2024). Zinc finger transcription factor MtZPT2-2 negatively regulates salt tolerance in *Medicago truncatula*. Plant Physiol..

[60] Kodaira K.S., Qin F., Tran L.S., Maruyama K., Kidokoro S., Fujita Y., Shinozaki K., Yamaguchi-Shinozaki K. (2011). Arabidopsis Cys2/His2 zinc-finger proteins AZF1 and AZF2 negatively regulate abscisic acid-repressive and auxin-inducible genes under abiotic stress conditions. Plant Physiol..

[61] Huang X.Y., Chao D.Y., Gao J.P., Zhu M.Z., Shi M., Lin H.X. (2009). A previously unknown zinc finger protein, DST, regulates drought and salt tolerance in rice via stomatal aperture control. Genes Dev..

[62] Zhang Y., Lan H., Shao Q., Wang R., Chen H., Tang H., Zhang H., Huang J. (2016). An A20/AN1-type zinc finger protein modulates gibberellins and abscisic acid contents and increases sensitivity to abiotic stress in rice (*Oryza sativa*). J. Exp. Bot..

[63] Chen J.H., Jiang H.W., Hsieh E.J., Chen H.Y., Chien C.T., Hsieh H.L., Lin T.P. (2012). Drought and salt stress tolerance of an Arabidopsis glutathione S-transferase U17 knockout mutant are attributed to the combined effect of glutathione and abscisic acid. Plant Physiol..

[64] Love M.I., Huber W., Anders S. (2014). Moderated estimation of fold change and dispersion for RNA-seq data with DESeq2. Genome Biol..

